# Testing the limits of FT-Raman spectroscopy for wine authentication: Cultivar, geographical origin, vintage and terroir effect influence

**DOI:** 10.1038/s41598-019-56467-y

**Published:** 2019-12-27

**Authors:** Dana Alina Magdas, Bogdan Ionut Cozar, Ioana Feher, Francois Guyon, Adriana Dehelean, Simona Cinta Pinzaru

**Affiliations:** 1 National Institute for Research and Development of Isotopic and Molecular Technologies, 67-103 Donat Str., 400293 Cluj-Napoca, Romania; 2 Service Commun des Laboratoires, 3 Avenue du Dr. Albert Schweitzer, 33608 Pessac, France; 3 Babes-Bolyai University, Biomolecular Physics Department, Kogalniceanu 1, RO-400084 Cluj-Napoca, Romania

**Keywords:** Biophysics, Chemistry

## Abstract

FT-Raman spectroscopy represents an environmentally friendly technique, suitable for the analysis of high-water content food matrices, like wines, due to its relatively weak water bending mode in the fingerprint region. Based on metabolomics applied to FT-Raman spectra, this study presents the classifications achieved for a sample set comprising 126 wines, originated from Romania and France, with respect to cultivar, geographical origin and vintage. Cultivar recognition was successfully performed among four varieties (Sauvignon, Riesling, Chardonnay, Pinot Gris) while subtle particularities exiting between the Chardonnay wines, coming from the two countries, because of terroir influences were pointed out. The obtained separations of 100% in both initial and cross-validation procedure for geographical differentiation between the two origin countries, as well as, among the three Romanian areas (Transylvania, Muntenia and Moldova) were also discussed. Apart of this, the limitations and the importance of choosing a meaningful data set, in terms of representativity for each classification criterion, are addressed in the present work.

## Introduction

During the last years, the development of reliable approaches, based on metabolomics, which can be successfully applied in food and beverages authenticity control, had gained a very rapid development^1–5^. The basic approaches used in metabolomics are represented by targeted and untargeted analysis (metabolic profiling and metabolic fingerprinting). Targeted methods are based on the concentration or signal intensity knowledge of interest analytes, while the aim of untargeted methods is to analyse as many signals as possible within a system, in an unsupervised manner. Subsequently, the obtained results are processed using statistical treatments, in order to look for sample patterns. Untargeted metabolic profiling is focused either on the study of a related metabolites group or to a specific metabolic pathway. The metabolic fingerprinting does not aim to identify all metabolites, rather to compare the patterns of metabolites among samples^4^. The untargeted methods are used in corroboration with an appropriate supervised chemometric technique, with the main aim to classify samples according to the defined classes.

Nowadays, an enhanced interest in the development and applications of analytical techniques which are reliable, rapid, cost-effective and more environmentally friendly is constantly manifested by different research groups. In order to implement an environmentally friendly method, the first step is to partially or totally reduce the wastes from the preparation step. This condition could be fulfilled through the direct analysis of the samples. This approach is not always possible, because of the specificity of each analytical method, but one of the techniques that can accomplish this condition is represented by Raman spectroscopy. From these considerations, we strongly believe that Raman spectroscopy, which represents nowadays an emerging technique in food and beverages authenticity studies, will benefit from an enhanced interest in the near future.

Raman technique is based on inelastic scattering of monochromatic light (laser beam) by molecules of samples. Most of the scattered light is elastically scattered (Rayleigh scattering), having a frequency which is equal to the frequency of incident radiation. Only a small fraction of scattered light of different frequency from incident light is comprised in Raman scattering. Usually, anti-Stokes-Raman scattering is generally weaker than Stokes-Raman scattering because most molecules are initially in their ground state. In the case of wine, a meaningful signal in the anti-Stokes region of the spectra was also obtained. The relatively high concentration of ethanol in wine could limit the interest of this technique because of ethanol dominant Raman signal that could hide weaker Raman signals from other molecules of interest presented in low amounts in wines composition. Nevertheless, our previous pilot study^6^, demonstrated that the intensities of ethanol peaks, which dominates Stokes region does not prevail in wide coverage of the anti-Stokes spectral range. Moreover, the importance of anti-Stokes spectral range for wine differentiation was emphasized in this previous study, performed on a sample set formed by 34 wines from Romania and France^6^. In this regard, the present work intends to prospect the classification efficiency and limitations of this approach, when an extended sample set, comprising 126 wine samples from Romania (80) and France (46), belonging to four cultivars (Sauvignon, Riesling, Chardonnay and Pinot Gris) and six vintages, was tested. Apart of this, the conditions that must be fulfilled, in terms of data set representatives, for successful classifications are discussed here.

## Materials and Methods

### Sample collection

In this study, a wine set, comprising 126 samples, was used and the sample distribution was listed in Table 1. The wines involved in this study were elaborated in laboratory, from grape samples and thus, giving us the control concerning the sample authenticity. A balanced sample distribution, in accordance with the classification criteria which were followed in this study, was chosen. This sample selection allowed the application of multidimensional data treatment for: (i) geographical origin differentiation (Romanian wines vs. French wines); (ii) cultivar recognition (all samples independent of geographical origin or vintage) and (iii) vintages separation (all cultivars from both countries).

**Table 1:** Sample repartition according to geographical origin, cultivar and vintage.

Sample distribution	Cultivar	Vintages						
		2011	2012	2013	2014	2015	2016	2017
Romania 80	Chardonnay 13	—	3	2	3	2	3	—
	Pinot Gris 9	—	2	2	2	1	2	—
	Riesling 20	—	5	4	5	2	4	—
	Sauvignon 38	3	8	8	8	6	5	—
France 46	Chardonnay 18	—	4	7	—	2	—	5
	Sauvignon 28	—	10	10	—	2	—	6

### FT-Raman measurements

Fourier transform Raman spectroscopy (FT-Raman) spectra were collected with a Bruker Equinox 55 FT-IR spectrometer with an integrated FRA 106S Raman module. A Nd:YAG laser emitting at 1064 nm, with the output power of 350 mW was used for FT-Raman spectra excitation. Spectra were acquired with 500 accumulations and spectral resolution of 4 cm^−1^. For Raman analysis 2 ml of each wine sample has been employed. A quartz cuvette with cap has been used for wines measurements under similar conditions of laser exposure and acquisition parameters as indicated. OriginPro 8.5 software (https://www.originlab.com/) was used for the processing of spectral data.

### Statistical data processing

The most widely employed supervised statistical method for classification purposes is linear discriminant analysis (LDA). In this case, the chemometric processing of experimental data was made using SPSS Statistics 24, IBM, USA (https://www.ibm.com/products/spss-statistics). The algorithm is based on finding linear combination among analysed variables, which can separate the predefined classes of samples. With these combinations a model is obtained, which is usually validated using “leave-one-out” cross validation method. This method takes out individually each sample and tries to reclassify it as a new one. The accuracy of the model is evaluated through the percent of correctly classified samples^7–9^.

### Ethical approval

This article does not contain any studies with human participants or animals performed by any of authors.

## Results and Discussion

**Figure 1:**
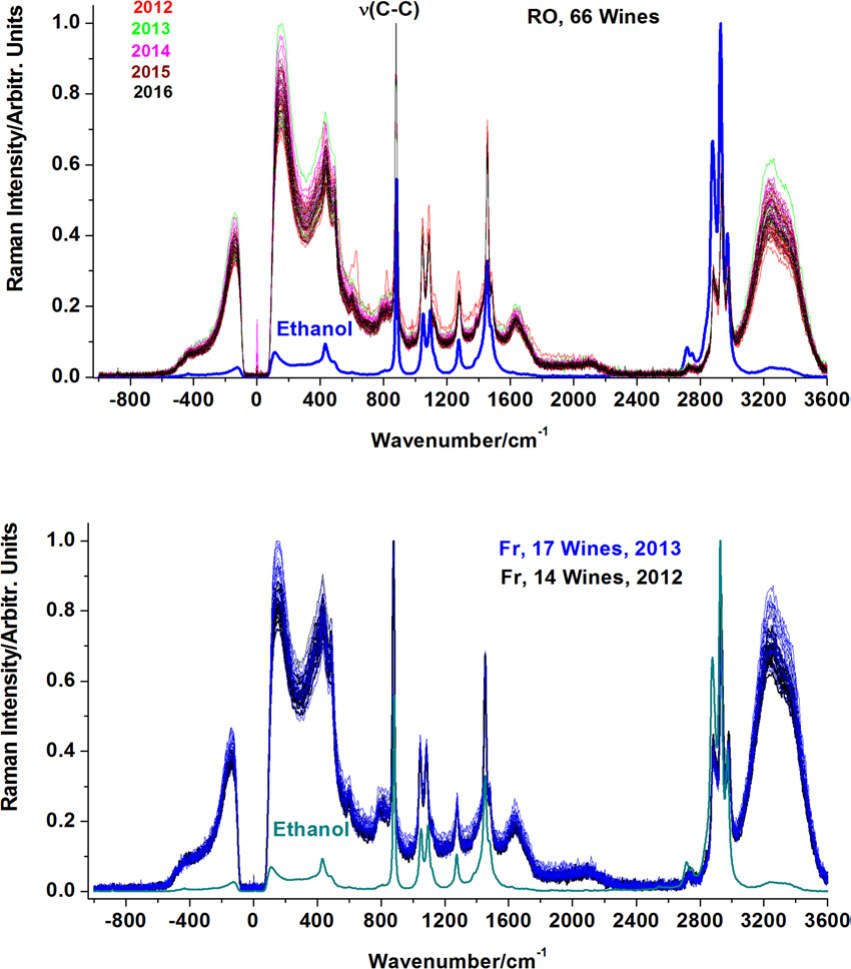
Normalized Raman spectra of Romanian (upper) and French (lower) wines. Excitation: 1064 nm, 350 mW. Note their variability regarding relative ratio of water-ethanol content, according to the ν (C–C) band intensity of ethanol and water (above 3200 cm^−1^).

**Figure 2:**
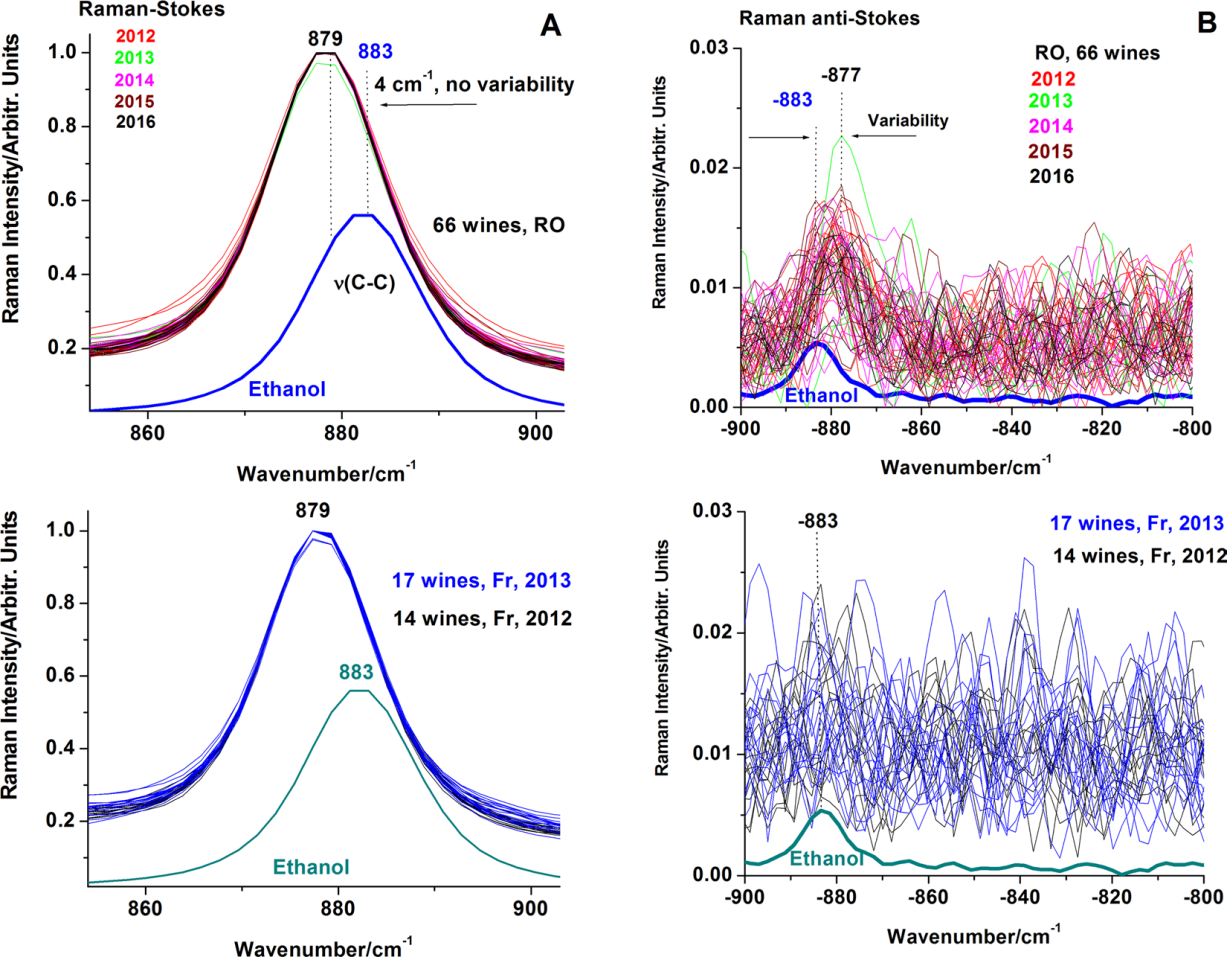
Spectral details of the 800–900 cm^−1^ Raman range in Stokes (**A**) and anti-Stokes (**B**) counterpart, evidencing the larger dispersed signal in anti-Stokes range and higher relative intensity, particularly for Romanian wines.

Raman spectra of all investigated wine samples were recorded in the spectral range to comprise anti-Stokes (−1000–0 cm^−1^) and Stokes (0–3600 cm^−1^) domains. Figure 1 shows the normalized -to-unit FT-Raman spectra of the investigated wines set. Each wines group (year) is plotted by individual color. Their overlap is comparatively displayed with the ethanol spectrum as indicated. We further examined in detail the specific spectral ranges showed in the Fig. 2, both in Raman-Stokes and anti-Stokes range. The normalized spectra of Romanian and French wines have been comparatively plotted in the 800–900 cm^−1^ range, where the strong ethanol ν(C-C) stretching mode occurs. Its position in the Stokes range is similar for all the studied wines at 879 cm^−1^ (Fig. 2), being shifted with about 4 cm^−1^ from its corresponding from pure ethanol (883 cm^−1^). Surprisingly, in anti-Stokes counterpart range, presented in detail in Fig. 2 this band showed a pronounced dispersive variability, particularly for the Romanian samples, while those of French origin lack a visible tendency in anti-Stokes. These features raised particular attention in considering the anti-Stokes range along with the Stokes 0–1700 cm^−1^ for chemometric analysis of Raman data.

The displacements of this signal in wines, as compared with those from pure ethanol is around 4 cm^−1^ (Fig. 2) in Stokes region while, in anti-Stokes, these shifts present a spreading of about 10 cm^−1^, suggesting that the interactions between molecular metabolites and the dominant water-ethanol solvent is specific for each wine, as a result of wine molecular specificity.

### Cultivar differentiation

**Figure 3:**
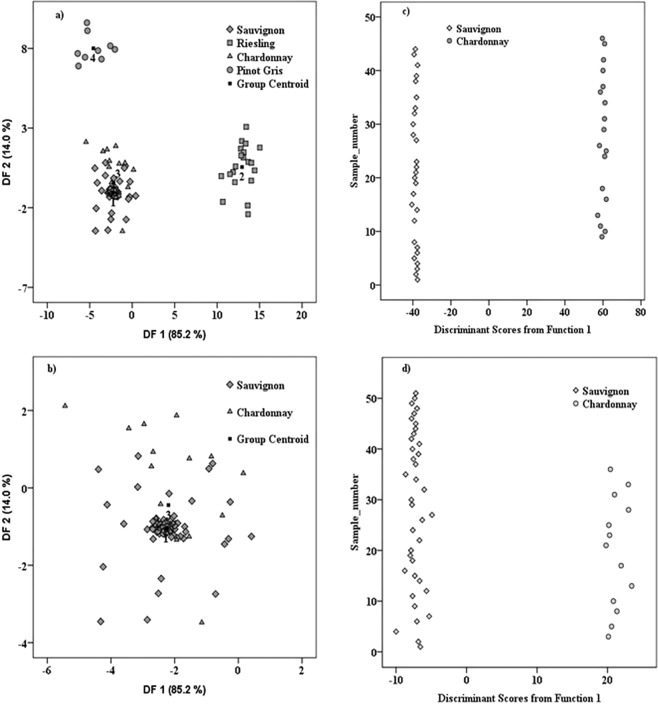
(**a**) Distribution of wine cultivars among the 126 samples from Romania and France; (**b**) Zoom on the overlapping area of Chardonnay and Sauvignon groups; (**c**) Chardonnay vs. Sauvignon (only French samples); (**d**) Chardonnay vs. Sauvignon (only Romanian samples).

For the variety discrimination, four white wine sorts were compared: Sauvignon-66, Riesling-20, Chardonnay-31 and Pinot Gris – 9 samples. From Chardonnay and Sauvignon cultivars, the analysed samples were originated from Romania and France, while those from Riesling and Pinot Gris varieties were only from Romania. As could be seen in Fig. 3a, a very good differentiation among Riesling, Pinot Gris and the group formed by Chardonnay and Sauvignon cultivars was achieved. A strong overlap between Chardonnay and Sauvignon samples was observed, thus making the differentiation of these two cultivars very difficult (Fig. 3b). This overlap was directly reflected into the obtained discrimination percentages. Thus, the simultaneous separation among the four cultivars conducted to an initial classification of 84.1%, while in cross-validation a modest percent of 74.6% was obtained. As four classes (corresponding to each cultivar) were used, the discrimination was made based on three functions: DF1 = 85.2%, DF2 = 14.0% and DF3 = 0.8%, which could explain the achieved classification.

The most important function, based on which, this differentiation is realized (DF1) comprises, among the main loadings, signals from: 1342; 1317; 1247; 1002; 522 and −464 cm^−1^. Some of these predictors (−464; 522; 1002; 1342 cm^−1^) were found to be discriminators for cultivars recognition in our earlier study, which successfully prospected the FT-Raman potential for wine authenticity^6^. The signal from 1247 cm^−1^ was among the differentiation markers for cultivar classification, in the study that applied SERS (Surface Enhanced Raman Spectroscopy) for white wine differentiation^10^. A new powerful identified marker here, is represented by the signal from 1317 cm^−1^, attributed in the literature to sinapic acid^11^.

The modest separation percentage obtained for the simultaneous cultivar classification, is due to the overlapping between the Sauvignon and Chardonnay samples. An interesting observation here, is that the two cultivars which cannot be discriminated are those coming from both countries (Romania and France) while, the varieties that were completed separated were formed only by Romanian samples. This observation conducts to the hypothesis that, the geographical influence partially overshadows the cultivar one. Thus, in order to verify our hypothesis and to find a way to differentiate the two wine varieties (i.e. Sauvignon vs. Chardonnay) an individual classification of these two sorts was proposed for each country.

In the first step, only the French samples (Sauvignon - 28 and Chardonnay - 18) were used for the classification. Thus, a percentage of 100% was recorded in both initial and cross-validation procedures (Fig. 3c). Among the predictors, that allowed this classification, the most powerful marker was the signal around −696 cm^−1^. This result is in agreement with the previous pilot study^6^ in which, the same discrimination marker was also found to have statistical significance for the differentiation among the three wine varieties (Sauvignon, Chardonnay and Feteasca). In the second step, the discrimination between the same two cultivars (Sauvignon - 38 vs. Chardonnay - 13) was also performed only on Romanian wines, in the attempt to verify the discrimination degree between above mentioned varieties. In this case, the separation between the two groups was made in a percentage of 100% in both initial and cross-validation classifications (Fig. 3d).

**Figure 4:**
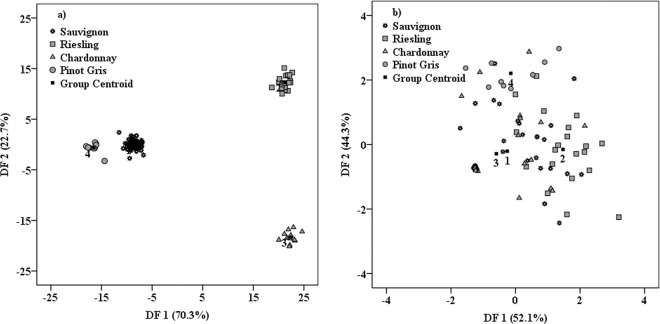
(**a**) Differentiation of the four cultivars after exclusion of French Chardonnay wines (108 samples); (**b**) Classification of investigated cultivars after exclusion of French Sauvignon wines (98 samples).

Taking into account that after the exclusion of geographical influences through performing separate classifications on every country, a perfect discrimination was achieved, we tried to better understand the concurrence between the varietal and geographical fingerprint by performing other two classifications. In the first classification we excluded all French Chardonnay samples and we differentiate among all remaining samples (108 samples). In this case, an excellent cultivar discrimination was obtained: 100% in initial classification and 99.1% in cross validation (Fig. 4a). Only one Sauvignon sample was misclassified being attributed to Pinot Gris group. From the second classification, French Sauvignon samples were excluded and all the other samples (98 samples) were kept for subsequent cultivar differentiation. A modest discrimination percentage was achieved in this case, 54.1% in initial and 49.0% in cross validation (Fig. 4b) because of Chardonnay samples, originated from both countries (Romania and France), which were impossible to be clustered into one single group. The explanation behind the weaker grouping abilities of Romanian and French Chardonnay wines is given by the versatility of this specific cultivar, which reflects combined effect of terroir components, as compared with other straightforward wines like Sauvignon^12^.

All these results demonstrate the applicability of FT-Raman spectroscopy in wine cultivar differentiation, even in very subtle cases such as those of Chardonnay cultivar, which is more sensitive to terroir compared to other wines (i.e. Sauvignon). This inconvenient could be overcome by choosing an appropriate reference data set, for each cultivar classification.

### Geographical discrimination

In previous reported study, made on 34 wine samples^6^, using the combination between FT-Raman technique and statistically treatment SLDA for geographical differentiation (Romanian vs. French wine samples) a classification of 100% in both initial and cross-validation procedures was achieved. The validation of our previous findings, was made in the present work, by using an extended sample set formed by 126 samples (Romania - 80 and France - 46), containing more cultivars, from distinct geographical regions. Also, this wine distribution offered us the possibility to follow the terroir effect which was also discussed in this section.

**Figure 5:**
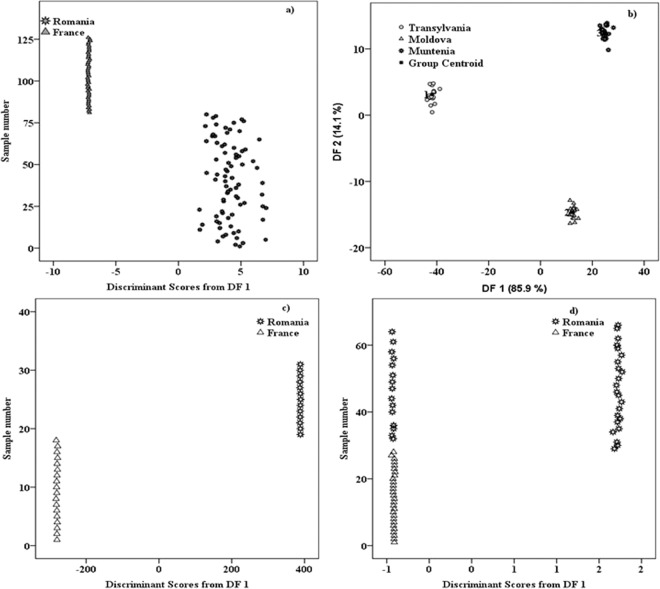
Geographical differentiation: (**a**) Romanian vs. French wine samples; (**b**) Among Romanian geographical regions (Transylvania, Moldova, Muntenia); (**c**) Terroir influence on Chardonnay cultivar; (**d**) Terroir effects on Sauvignon wines.

The experimental data processing allowed a perfect separation (100% in initial and cross-validation procedure), between samples from Romania and France (Fig. 5a). The main predictors that allowed the present classification were: −451, 1453, −455, 503, 1407, 1428 and 1457 cm^−1^. According to previously published work^11^, ferulic and sinapic acids give peaks around 1457 cm^−1^, while the predictor from 503 cm^−1^ might be attributed to an overlapped signal of sinapic acid^13^ and glucose^14^.

The potential of this approach, to differentiate wines coming from much closer geographical areas (i.e. inside the same country) was also tested in this work. For this purpose, the wine classification, with respect to the three Romanian regions, namely (Transylvania, Moldova and Muntenia), was successfully performed and the achieved percentage was 100% in both classifications (initial and cross-validation) (Fig. 5b). The classification was made based on two functions, which contributed to this differentiation as follows: DF1:85.9% and DF2:14.1%, respectively. First function DF1 comprises its most powerful coefficients loadings from 1484 and 1172 cm^−1^, while the main predictors contained in DF2 are represented by the signals from 1230 and 777 cm^−1^. Among all these predictors, only one could be tentatively assigned according to the literature^11^, which is the signal from 1172 cm^−1^ that belongs to caffeic acid. From anti-Stokes region, the most powerful markers that allowed this classification were those located at −571, −611, −667 cm^−1^. Apart of these, some predictors (−709, −721 and −503 cm^−1^), which were found in our previous study^6^ to be effective in geographical discrimination, were now identified among the predictors that allowed this classification, but without having a major statistical significance. The main explanation, for having in this classification other main discriminant loadings, is given by the fact that between our previous study and the present one, there are only two common geographical areas. In the first study, the geographical classification was made among Romanian regions: Transylvania, Moldova and Banat, while in present work the third production area was Muntenia instead of Banat.

Terroir influence of Chardonnay and Sauvignon cultivars coming from both countries, was also followed in this study. In this regard, a differentiation of the same wine cultivar, produced in Romania versus its similar from France was attempted (Fig. 5c,d). A total separation is observed for the Chardonnay samples (100% in both initial and cross-validation procedure) while for Sauvignon cultivar a separation of 77.1% in both classifications was achieved. These classifications sustained our previous supposition according to which Chardonnay cultivar appear to be more sensitive to the combined effects of terroir as compared with Sauvignon. Nevertheless, extended studies implying more wine cultivars and very distinct geographical origins should be performed before embracing a conclusion concerning the terroir influence on cultivar specificities.

### Vintage differentiation

For vintage discrimination, Romanian and French samples, from all cultivars, were separately classified, inside of each country. This because, in this case, the differences observed among wines coming from distinct vintages are given by the meteorological conditions that prevailed in every specific year. From the very beginning, it can be stated that, due to the important variability of meteorological conditions of these two countries (Romania and France) a comparison of all wine samples implied in this study would be meaningless.

**Figure 6:**
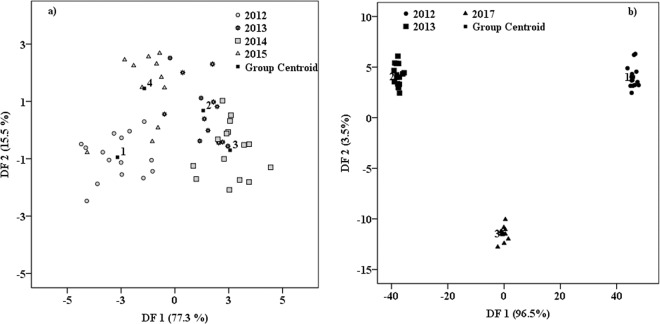
(**a**) Vintages (2012, 2013, 2014, 2015) discrimination of Romanian wines; (**b**) Vintages (2012, 2013, 2017) discrimination of French samples.

Starting from these considerations, the first proposed differentiation was among the Romanian wines (from all cultivars) coming from five consecutive vintages (2012–2016). For this simultaneous differentiation, a very low classification percent was reached, 38.5% for initial classification and 33.8% in cross-validation. The main reason that conducted to this totally unsatisfactory differentiation percentage was because, the samples produced in 2016 vintage, overlapped the other wines of 2012–2015. By excluding 2016 vintage from the classification (Fig. 6a), the discrimination percentage strongly improved (90.7% in initial classification and 77.8% in cross-validation). This classification was obtained based on three functions, that can explain the total variation as follows: DF1 (77.3%), DF2 (15.5%) and DF3 (7.3%). The loadings based on which this classification was achieved were located in both spectral regions (Stokes and anti-Stokes), but those having a stronger prediction capability were located in Stokes region. The main predictors of DF1 were the signals from: 804, 985, 960 and 1635 cm^−1^, that were attributed in the literature to caffeic (804 cm^−1^), caftaric (985, 1635 cm^−1^) and ferulic acids (960 cm^−1^)^11,13,15^. Finding caftaric acid the most powerful marker for production year could be explained by the compound being directly related to oxidation levels through the wines undergone^3^, therefore being dependent on wine aging.

The vintage differentiation, performed for French wine samples, was made using samples from three distinct production year (2012, 2013 and 2017) and was successfully achieved in a percentage of 100% in both initial and cross-validation procedure (Fig. 6b). This classification was made based on two discriminant functions which contributed to the present vintage differentiation as follows: DF1 (96.5%) and DF2 (3.5%). The main predictors comprised in DF1 are the signals from −848, −850, 492, 601, 794, 1022 and 1240 cm^−1^. According to the literature, some of these signals might be associated as follows: 492 cm^−1^- ferulic acid^13^, 1022 cm^−1^ – sinapic acid^11^ and 1240 cm^−1^ an overlapped signal due to p-coumaric acid and caftaric acid^11,13^.

## Conclusions

The present work proposed a versatile method through which, by using the same analytical method and statistical treatment, different kind of wine classifications can be achieved (cultivar, geographical origin and vintage). In this context, it was also confirmed the potential of anti-Stokes spectral region, along with fingerprinting region from Stokes area, for white wine discrimination studies, based on FT-Raman metabolomics. Using the proposed approach, the successful differentiation of investigated cultivar (Sauvignon, Chardonnay, Riesling and Pinot Gris) was underlined as well as the limitation that should be considered in regard to the distribution of reference dataset, according to the classification purpose. The sensitivity of FT-Raman technique, in conjunction with chemometrics, was proved, even for underlying subtle differences that are exiting between the Chardonnay wines coming from the two countries (Romania and France) that might be due to a terroir effect.

For the geographical classification of studied wines, a 100% in initial and cross-validation classification was obtained for the differentiation of Romanian and French wines, as well as for the samples coming from the three Romanian geographical regions. Concerning vintage discrimination, a 100% percentage in initial and cross-validation was obtained for French samples coming from 2012, 2013 and 2017 vintages, while for simultaneous classification of the four consecutive Romanian vintages 2012–2015, a percentage of 90.7% in initial classification and 77.8% in cross-validation was achieved, after the exclusion of 2016 vintage.

From all these conclusions it could be stated that, for the successful wine discrimination, based on FT-Raman metabolomics, a meaningful experimental data set, in terms of sample number and repartition among classes, should be carefully chosen, for each classification, in order to assure the representativeness of reference data.
